# Editorial: Understanding plant diversity and evolution in the Mediterranean Basin

**DOI:** 10.3389/fpls.2023.1152340

**Published:** 2023-02-14

**Authors:** Gonzalo Nieto Feliner, Nico Cellinese, Andrew A. Crowl, Božo Frajman

**Affiliations:** ^1^ Department of Biodiversity and Conservation, Real Jardín Botánico (RJB), CSIC, Madrid, Spain; ^2^ Florida Museum of Natural History, University of Florida, Gainesville, FL, United States; ^3^ Department of Biology, Duke University, Durham, NC, United States; ^4^ Department of Botany, University of Innsbruck, Innsbruck, Austria

**Keywords:** Mediterranean Basin, systematics, evolution, biogeography, phylogeography, phylogenetics, hybridization, polyploidy

The biota of the Mediterranean Basin (MB), characterized by a high species richness and endemism (Greuter, 1991; Blondel et al., 2010), has drawn the attention of biologists for centuries. While representing only 1.6% of Earth’s surface area, this hotspot of biodiversity houses 7–10% of the world’s plant diversity, 60% of which is restricted to the region (Myers et al., 2000; Thompson, 2005). Two main factors which have been shown to be important for high diversity in other Mediterranean climate zones–characterized by mild wet winters and warm dry summers– are low rates of extinction and an association with fire (Rundel et al., 2018). However, many additional features and forces shape biodiversity patterns in the MB (e.g., Nieto Feliner, 2014; Onstein et al., 2015).

Abiotic factors, in particular a complex and active geo-climatic history (Krijgsman, 2002; Rosenbaum et al., 2002), have played a crucial role in shaping the MB flora. Three climatic events are considered as fundamental historical landmarks in plant evolution within the region. These are the Messinian Salinity Crisis (MSC) c. 5.9 Ma (Bocquet et al., 1978), the advent of Mediterranean climate c. 3.2 Ma (Suc, 1984) and the climatic oscillations during the Pleistocene c. 2.5 Ma onwards (Hewitt, 2000). These abiotic events have been tested as drivers of diversification in numerous phylogenetic studies (e.g., Fiz-Palacios and Valcárcel, 2013; Crowl et al., 2015). However, in a biota as rich and diverse as that found in the MB, an accurate picture of diversification drivers across taxonomic groups and geographic areas requires consideration of additional factors. These include evolutionary consequential mechanisms such as polyploidy and hybridization (Thompson, 2005; Marques et al., 2018), the importance of annual habit and associated selfing reproduction (Stebbins, 1970), the heterogeneous orography that provided refugial areas in periods of adverse climates during the Pleistocene (Médail and Diadema, 2009), and the structure of the landscape, composed of a mosaic of habitat patches (Blondel et al., 2010). This structure could partly underlie features such as the importance of chasmophytic species (Davis, 1951), the role of ecological speciation (Thompson et al., 2005), in addition to shaping distribution limits of narrow endemics, which are highly represented in the MB (Thompson, 2005).

Another crucial factor that greatly influenced the MB biota across time is human impact. Flanking the fertile crescent, cradle of western civilizations, the impact of humans on the MB over, at least, the last 10,000 years has probably been the most intense on the planet leading to landscape modification, habitat destruction and species range fragmentation (Blondel et al., 2010; Thompson, 2020). In addition, the MB acted as a corridor for the rapid spread of useful wild species such as chestnut (Fineschi et al., 2000) or stone pine (Vendramin et al., 2008) and domesticated species or varieties (Zohary et al., 2012) although not always along an expected straight east-to-west direction, but rather involving the preservation of ancestral local lineages preceding agriculture (Besnard et al., 2018; Baumel et al., 2022).

Despite a considerable body of knowledge on plant evolution in the MB, we still have an insufficient understanding about the various potential drivers of diversification that, over time, have interacted with other numerous intervening factors to produce the rich plant biodiversity we observe. This is no doubt partly due to the complexity of the evolutionary history in the basin (Nieto Feliner, 2014). The present special issue is a reflection of the broad array of research foci and methodological approaches that the scientific community use to tackle the fascinating questions related to plant evolution in the MB.

Eight of the fifteen papers included in this special issue adopt macroevolutionary perspectives attempting to understand diversification patterns, taxonomic circumscriptions, and biogeographic connections of species lineages occurring in the MB and tested under explicit phylogenetic and phylogenomic frameworks. One of the factors generating uncertainty in taxonomy and phylogenetic relationships is polyploidy, which is documented in five of these studies. Cetlová et al. (2021) shows a combination of representative features for the MB in *Alyssum*, ranging from annual lifespan to multiple events of allopolyploidy, but also highlighting a strong human influence shaping its current distribution, and an impact of range shifts caused by sea-level changes, resulting in complex patterns. The same complexity is found in the *Euphorbia nicaeensis* alliance, which, as several other plant groups (e.g., Manafzadeh et al., 2014), colonized the MB from Western Asia and the Irano-Turanian region, and owes its partly unsettled taxonomy —including cryptic diversity— to diversification driven by vicariance in three main European Pleistocene refugia, ecological adaptation and, to a lesser extent, polyploidy (Stojilkovič et al., 2022). Polyploidy often evolves independently in different groups (Segraves et al., 1999). The following two studies identified different polyploidization patterns across the MB over time. Tomasello and Oberprieler (2022) find that elevational range shifts and secondary contact fostered allopolyploidy in Iberian populations of the genus *Leucanthemopsis*, whereas in other parts of its Mediterreanean-Alpine range, only autopolyploidy occurred. In the genus *Centaurium*, with tetraploids mainly distributed in northern temperate areas and hexaploids in southern arid areas, Maguilla et al. (2021) infer through diversification estimates, biogeographic analysis and chromosome evolution, that diploid lineages remained in the area of origin. By contrast, whole genome duplication events —recent and old— could have facilitated colonization and establishment in other areas. Aiming to tackle the complexity derived from polyploidy, Slenker et al. (2021) developed a new phylogenomic approach to identifying merged subgenomes from different progenitors, which allow them to infer an allopolyploid origin for the Greek endemic *Cardamine barbaraeoides*. Naciri et al. (2022) conclude that the chasmophyte habit, important in the MB, evolved independently several times in *Silene* sect. *Italicae*. The remaining two phylogenetic studies find high diversification rates: first, in the Iberian-centered genus *Antirrhinum*, associated with a geographic mode of speciation and multiple acquisition of key taxonomic characters (Otero et al., 2021); and second, in the Mediterranean lineage of *Limonium*, associated with the MSC, the onset of Mediterranean climate, Plio-Pleistocene sea-level fluctuations, and apomixis (Koutroumpa et al., 2021).

In line with the diversity of possible evolutionary outcomes, hybridization can be addressed under different approaches. The contribution by López de Heredia et al. (2021) exemplifies how in two hybridizing species of the best known syngameon —*Quercus* (Cannon and Petit, 2020)— cytonuclear incompatibilities may have shaped the combination of plastid and nuclear genomes across the species ranges. Abdelaziz et al. provide evidence of a stable unimodal hybrid zone in the genus *Erysimum*, one of the possible evolutionary outcomes of hybridization, which however has been insufficiently studied in plants (Abbott, 2017).

One of the unwritten goals of the present special issue was to enrich our knowledge concerning the Balkan Peninsula, a critical area for the evolution of MB biota, as well as for the postglacial recolonization of central Europe (Hewitt, 2011). In addition to Slenker et al. (2021), four other papers focused on this region. Rešetnik et al. (2022) and Torre et al. (2022) unveil the role of the Balkans as a cradle of diversification and a source for dispersal into other areas in *Aurinia saxatilis* and *Ulmus laevis*, respectively. Lazarević et al. (2022) explore the consequences of interploidy hybridization in the areas of contact between two Tertiary relict species of the long-lived genus *Ramonda* using AFLPs and genome size estimation. Using an integrative approach that includes environmental modelling, Faltner et al. (2023) provide clues to the causes behind very narrowly-distributed endemics in the MB and clarify the unforeseen phylogenetic position of one of them. Focusing on *Euphorbia orphanidis*, an endangered species restricted to a few patches of limestone screes in Mt. Parnassos (Greece), this study underlines the role of microrelief in heterogeneous mountain environments. Finally, a phylogeographic study focuses on a narrow endemic coastal species, *Eokochia saxicola*, which is exceptional in the MB for being wind-pollinated, and concludes that, despite small population sizes, this rare species does not face immediate conservation risks (Strumia et al., 2021).

The Mediterranean Basin is undoubtedly a remarkable biogeographic region with a rich but fragile flora. We hope that this collection of studies provides the inspiration and motivation for much needed additional research in this biodiversity hotspot.

## Author contributions

All authors listed have made a substantial, direct and intellectual contribution to the work, and approved it for publication.
